# Analysis of Clinical Features of Kounis Syndrome Induced by Cephalosporin

**DOI:** 10.3389/fcvm.2022.885438

**Published:** 2022-04-26

**Authors:** Weijin Fang, Liying Song, Zhenzhen Deng, Wei Sun, Zuojun Li, Chunjiang Wang

**Affiliations:** Department of Pharmacy, The Third Xiangya Hospital, Central South University, Changsha, China

**Keywords:** Kounis syndrome, coronary artery spasm, cephalosporin, chest pain, allergic angina

## Abstract

**Background:**

Cephalosporins are an increasingly encountered cause of Kounis syndrome. The present study examined the clinical features of cephalosporin-induced Kounis syndrome and provided references for diagnosis, prevention, treatment, and prognosis.

**Methods:**

We collected cephalosporin-induced Kounis syndrome case reports by searching Chinese and English databases from the establishment of the database to October 31, 2021.

**Results:**

Twenty-five patients (17 males and eight females) were included, with a median age of 61 years (range 33–92). Cephalosporins were administered via oral, intravenous and intramuscular routes. All reactions occurred within 30 min, except in two patients. Fourteen patients experienced chest pain, 19 experienced hypotension, 16 had cutaneous reactions, 10 had respiratory symptoms, and seven had gastrointestinal symptoms. Thirteen patients had elevated troponin levels, and eight patients had elevated serum tryptase levels. The electrocardiogram showed ST-segment elevation in 13 patients, depression in four patients, and elevation and depression in six patients. Coronary angiography showed normal results in 12 patients and abnormal results in 13 patients. The skin prick test was positive for cephalosporin in three patients. Twenty-four of the 25 patients recovered after being given anti-allergic and acute coronary syndrome treatment, and there was one death.

**Conclusions:**

Kounis syndrome is a serious adverse reaction to cephalosporin. Clinicians should consider Kounis syndrome in every patient receiving cephalosporin and presenting with acute chest pain or anaphylactic symptoms.

## Introduction

Anaphylaxis is a severe, life-threatening, generalized or systemic hypersensitivity reaction (1). Cardiac tissue is susceptible to hypersensitivity processes (2). Myocardium, conduction system and coronary artery reactions to various allergens are well- established. The clinical condition of allergic angina syndrome was not described until 1991, as coronary spasm progresses to allergic acute myocardial infarction (3). Kounis syndrome (KS) is an acute coronary syndrome caused by an allergic reaction to foods, drugs, environmental exposures, and various conditions (4). KS is mediated by mast cells that interact with macrophages and T lymphocytes and results in the massive release of inflammatory mediators in cardiac tissue, coronary arteries and plaques (5). The main clinical signs and symptoms of KS are associated with allergic reactions accompanied by cardiac symptomatology. It is not a rare disease, but it is infrequently diagnosed. Although the incidence of KS is not clear, its special clinical manifestations and treatment have attracted clinical attention.

Antibiotics are the most common culprit for KS (6). Cephalosporins are commonly used antibiotics in hospitalized patients and outpatients. Hypersensitivity is the most common adverse reaction to cephalosporins (7). However, KS is a rare side effect of cephalosporins. Limited data are available for cephalosporin-induced KS. The present article collected relevant case reports to examine the cephalosporin types, clinical manifestations, electrocardiographic changes, laboratory abnormalities, and echocardiographic and angiographic findings of cephalosporin-induced KS. This research is of great significance to the diagnosis, treatment, prognosis and prevention of cephalosporin-induced KS. This research also provides a basis for clinicians to improve their understanding and diagnosis of KS.

## Materials and Methods

### Search Strategy

We searched databases from the establishment to October 31, 2021, including PubMed, Embase, The Cochrane Library, CNKI, VIP database and Wanfang database. The search method used a combination of subject words and free words, including Kounis syndrome, acute coronary syndrome, acute myocardial infarction, myocardial infarction, allergic angina syndrome, allergic angina, allergic myocardial infarction, vasospastic allergic angina, coronary artery disease, coronary spasm, coronary thrombosis, stent thrombosis, myocardial ischemia, chest pain, hypersensitivity, anaphylactic, anaphylactoid, antibiotics, cephalosporins (first-, second-, third-, fourth- and fifth-generation cephalosporins listed), beta-lactams, and adverse reactions.

### Data Extraction

We used a self-designed table to extract relevant information of the patient, including country, sex, age, underlying disease, combined medication, cephalosporin administration, clinical manifestations, laboratory examinations, imaging examinations, treatment and prognosis.

## Results

### Basic Information

We initially identified 832 studies. Two hundred and ninety seven replicate studies were excluded. After an initial screening of titles and abstracts, a total of 467 articles were removed. Of the remaining 68 studies, a total of 25 articles were included after full-text screening (8–32). Patient information is summarized in Table 1. Twenty-five patients (17 males and eight females) were primarily from Europe (seven from Turkey), with a median age of 61 years (33–92 years). Cephalosporins were primarily used for perioperative antibiotic prophylaxis (13 patients) and infection treatment (nine patients). The cephalosporins included cefuroxime (seven patients), ceftriaxone (seven patients), cefazolin (five patients), cefoperazone-sulbactam (two patients), cefotaxime (one patient), cefoxitin (one patient), ceftazidime (one patient), and cefditoren (one patient). The route of administration included intravenous (18 patients), oral (two patients), intramuscular (two patients), and unknown (three patients). Thirteen patients had risk factors for KS, and 9 patients used other drugs simultaneously.

**Table 1:** Basic information of the 25 included patients.

**Reference**	**Region**	**Sex/Age**	**Coronary risk factors and underlying disease**	**Combination therapy**	**Indication**	**cephalosporin**	**Dose(g)**	**Route**	**Onset time**
Mazarakis et al. (8)	Greece	F/70	Hypercholesterolemia	Diazepam	AP: gynecological procedure	Cefuroxime	0.75	IV	1 min
Ilhan et al. (9)	Turkey	F/61	T2DM	NR	NR	CA	0.25	oral	10 min
Caglar et al. (10)	Turkey	F/85	Hypertension, MI	NR	HAP	Ceftriaxone	first dose	NR	30 min
Sánchez et al. (11)	Spain	F/58	Hypercholesterolemia	Ranitidine, ondansetron, midazolam, propofol, dexamethasone, fentanyl, atropine, rocuronium, paracetamol, dexketoprofen	AP: arthroscopically repair the rotator cuff	Cefazolin	2	NR	Immediate
Adachi et al. (12)	Japan	F/92	NR	Lidocaine	AP: bladder cancer surgery	Cefazolin	1	NR	5 min
Gao et al. (13)	China	F/37	NR	NR	AP: resection of sweat glands	Cefuroxime	1.5	IV	75 min
Ricciardi et al. (14)	Italy	F/73	Smoker, hypertension, dyslipidemia, hypothyroidism	NR	AP: cystoscopy	Ceftriaxone	NR	IV	Immediate
Sato et al. (15)	Japan	F/69	Hypertension	Propofol, rocuronium, fentanyl, desflurane, remifentanil	AP: TURBT and LLN	Cefazolin	NR	IV	NR
lgenli et al. (16)	Turkey	M/24	NR	NR	LRTI	Ceftriaxone	NR	IM	Immediate
Kitulwatte et al. (17)	Sri Lanka	M/52	Diabetes	NR	Trauma	ceftazidime	NR	IV	Immediate
Sequeira et al. (18)	Portugal	M/56	Dyslipidemia	Midazolam, fentanyl, rocuronium, propofol	AP: knee arthroscopy	Cefazolin	NR	IV	Immediate
Biteker et al. (19)	Turkey	M/90	NR	NR	UTI	CA	0.75	IM	10 min
Murat et al. (20)	Turkey	M/40	NR	NR	NR	CA	0.5	oral	5 min
Yurtdaş et al. (21)	Turkey	M/42	NR	NR	NR	Ceftriaxone	NR	IV	20 min
Saleh et al. (22)	Jordan	M/65	NR	NR	UTI	Ceftriaxone	1	IV	10 min
Barbarroja-Escudero et al. (23)	Spain	M/64	Smoker	Acetaminophen, acenocoumarol, clarithromycin	CAP	Cefditoren pivoxil	first dose	IV	6 h
Venkateswararao et al. (24)	India	M/36	NR	NR	pneumonia	cefotaxime	1	IV	5 min
Mitsis et al. (25)	UK	M/64	Hypertension, dyslipidemia, smoker	Diazepam	AP: bone reconstruction	Cefuroxime	0.5	IV	20 min
Absmaier et al. (26)	Germany	M/60	PH, hypertension, COPD, nicotine abuse	NR	AP: TURP	Cefuroxime	nr	IV	2 min
Çakmak and Keskin (27)	Turkey	M/33	NR	Metronidazole	Acute appendicitis	Ceftriaxone	nr	IV	30 min
Fujita et al. (28)	Japan	M/72	Hypertension, smoker	Propofol	AP: choledocholithiasis	CPS	1.5	IV	Immediate
Austin et al. (29)	USA	M/64	NR	NR	AP: radical neck dissection	Cefoxitin	2	IV	10 min
Ito et al. (30)	Japan	M/74	NR	NR	Acute appendicitis	CPS	1	IV	10 min
Forlani et al. (31)	Italy	M/61	Obesity, hypertension	NR	AP: saphenous vein stripping	Ceftriaxone	nr	IV	a few min
Mota et al. (32)	Portugal	M/56	NR	NR	AP: knee arthroscopy	Cefazolin	nr	IV	Immediate

*AP, antibiotic prophylaxis; CA, cefuroxime axetil; CAP, community-acquired pneumonia; CPS, cefoperazone-sulbactam; COPD, chronic obstructive pulmonary disease; EF, ejection fraction; F, female; HAP, hospital-acquired pneumonia; IV, intravenous; IM, intramuscular; PH, prostate hyperplasia; T2DM, type 2 diabetes mellitus; LRTI, lower respiratory tract infection; M, male; MI, myocardial infarction; TURBT, transurethral resection of bladder; UK, United Kingdom; USA, United States of America; UTI, urinary tract infection*.

### Clinical Manifestations

The clinical characteristics of the 25 included patients are summarized in Table 2. The time of administration and symptom onset varied from immediate to 6 h. Fourteen patients developed chest pain, and 19 patients developed hypotension. Allergic skin reactions occurred in 16 patients, including skin rash (15 patients) and itching (eight patients). Ten patients developed respiratory symptoms or signs, seven patients developed gastrointestinal symptoms, and nine patients developed neurological symptoms. One patient experienced cardiac arrest.

**Table 2:** Clinical manifestations and laboratory and imaging examinations of the 25 included patients.

**Reference**	**Clinical manifestations**	**Blood pressure (mmHg)**	**Troponin (ng/mL)**	**CK-MB (U/L)**	**Tryptase (mg/L)**	**ECG**	**ECHO**	**Coronary angiography**
Mazarakis et al. (8)	Retrosternal pain, rash, periorbital edema, itching, vomiting, pale	70/50	Normal	Normal	29	ST elevation	NR	Normal
Ilhan et al. (9)	Fatigue, nausea, vomiting, vertigo, chest pain, confusion, erythema, dyspnea	70/40	0.06	59	NR	ST elevation; ST depression	Inferior wall hypokinesia	Normal
Caglar et al. (10)	Itching, rash, skin lesions, chest pain, dyspnea	Hypotension	NR	↑	↑	ST elevation	Enlargement of LA and LV, LV systolic dysfunction, EF: 35%	Normal
Sánchez et al. (11)	Bronchospasm, rash, hypotension	63/39	NR	NR	66.4	ST elevation	NR	Normal
Adachi et al. (12)	Shortness of breath, nausea, shock, loss of consciousness	Unrecordable	NR	NR	26.7	ST elevation	NR	Normal
Gao et al. (13)	Nausea, vomiting, pale, vertigo, headache, heart failure, rash, itching	60/40	10.90	74.02	NR	ST depression	LV inferior wall hypokinesia, EF:26%	Normal
Ricciardi et al. (14)	Flushing, erythema, itching, chest pain	NR	Normal	Normal	NR	Acute MI	NR	Normal
Sato and Arai (15)	Rash, hypoxemia	70/-	Normal	Normal	NR	ST depression	Normal	Normal
Ilgenli et al. (16)	Chest pain, dyspnea, perturbed, redness in the face and eyes, hypotension	90/60	borderline^*^	NR	NR	ST elevation	NR	Ectasia in RCA
Kitulwatte et al. (17)	Chest pain, unconsciousness, dyspnea	Hypotension	NR	NR	118	NR	NR	30–40% atheroma
Sequeira et al. (18)	Hypoxemia	40/-	7	NR	NR	ST depression	LV systolic dysfunction	RCA spasm
Biteker et al. (19)	Chest pain, rash	Normal	22	85	43.5	ST elevation	Inferior wall hypokinesia	Plaques in LAD and CX
Murat et al. (20)	Chest pain	NR	Normal	Normal	NR	ST elevation	Normal	Plaques in CA and RCA
Yurtdaş et al. (21)	Chest pain, dyspnea, sweating, nausea, vomiting, urticarial, edematous lesions	75/40	3.7^*^	NR	29	ST elevation	NR	98% stenosis of proximal of RCA
Saleh et al. (22)	Epigastric pain, malaise, shortness of breath, chest pain, drowsiness, hypoxemia	80/50	1.6^**^	NR	NR	ST elevation	NR	Stenotic of RCA
Barbarroja-Escudero et al. (23)	Epigastric pain, vegetative symptoms	NR	1.32	8^a^	4.5	ST elevation, ST depression	Normal	Multivessel vasospasm
Venkateswararao et al. (24)	Iching, sweating, headache, chest pain, facial and periorbital swelling, hypotension	80/60	1.027^*^	NR	NR	ST elevation, ST depression	Normal	Normal
Mitsis et al. (25)	Acute bronchospasm, erythema, itching, periorbital edema, general discomfort, dizziness, chest pain, AF	80/50	0.266^**^	↑	NR	ST elevation, ST depression	Hypokinesia of IVS and apex of LV, EF:40–45%	Severe spasm of LMCA
Absmaier et al. (26)	Flush, dyspnea, chest pain, bitter taste, burning feeling	80/-	0.132 ^*^	NR	7.95	ST elevations	NR	Stenosis of RCA, vasospasm of coronary vessels
Çakmak and Keskin (27)	Chest pain, nausea, itching, rash	Normal	2.89	NR	NR	ST elevation, ST depression	Normal	Normal
Fujita et al. (28)	Tachycardia	60/42	NR	21	NR	ST elevation	Normal	Normal
Austin et al. (29)	Urticaria, tachycardia	40/-	NR	normal	NR	ST elevation, ST depression	Normal	Normal
Ito et al. (30)	VF, rash	58/36	NR	27	53.4	ST elevation	NR	stenosis of RCA
Forlani et al. (31)	Urticaria, loss of consciousness, cardiogenic shock	NR	16.2^**^	97^a^	NR	ST elevation	Hypertrophy of LV, hypokinesia of IVS and apex, EF:50%	Thrombosis of AICA
Mota et al. (32)	Anaphylactic shock, hypoxemia	Hypotension	7^**^	NR	NR	ST depression	Ventricular dysfunction and segmental alterations	Severe RCA spasm

*AF, atrial fibrillation; AICA, anterior interventricular coronary artery; CX, circumflex; DES, drug-eluting stent; ECHO, echocardiography; NR, not reported; IVS, interventricular septum; LAD, left anterior descending; LV, left ventricle; LMCA, left main coronary artery; MI, myocardial infarction; PCI, percutaneous coronary intervention; RCA, right coronary artery; VF, ventricular fibrillation*.

* *: Troponin T*;

** *: Troponin I or Troponin T*.

a *indicates that the unit of CK-MB is ng/ml*.

### Laboratory Examination

The laboratory test results are summarized in Table 2. Troponin levels were elevated in 13 of 18 patients, and creatine kinase-MB was elevated in nine of 14 patients. Serum tryptase was elevated in eight of 10 patients. The skin prick test was positive in three of seven patients. Intradermal tests were positive in six patients.

### Imaging Examination

The imaging examination results are summarized in Table 2. Electrocardiograms (ECGs) primarily showed ST elevation (13 patients), ST depression (four patients), and ST elevation and ST depression (6 patients). Echocardiography in 15 patients showed hypokinesia (five patients), left ventricular systolic dysfunction (three patients), and reduced ejection fraction (four patients). Coronary angiography in 13 patients primarily showed spasm (five patients), stenosis (four patients) and plaque (three patients).

### Treatment

The treatment and prognosis of the 25 included patients are summarized in Table 3. Drug treatment included corticosteroids (18 patients), antihistamines (15 patients), epinephrine (nine patients), vasodilators (16 patients), and antiplatelet drugs (nine patients). Two patients underwent cardiopulmonary resuscitation. Three patients underwent revascularization. Twenty-four patients eventually recovered, and one patient died. Twenty-one patients belonged to the type I variant, and 4 patients belonged to the type II variant.

**Table 3:** Treatment and outcome of the 25 included patients.

**Reference**	**Revascularization**	**Cardiac arrest**	**KS type**	**Prick-test/** **IDT**	**Treatment**	**Outcome**
Mazarakis et al. (8)	NR	NR	I	NR	Steroid, antihistaminic, morphine, ASA, CCB, nitroglycerin	Recovery, discharged after 4 d
Ilhan et al. (9)	NR	NR	II	NR	Epinephrine, clopidogrel, LMWH, statin, CCB	Recovery, discharged after 1 w
Caglar et al. (10)	NR	NR	II	NR	Steroid	Recovery
Sánchez et al. (11)	NR	NR	I	+/NR	Steroid, antihistaminic, nitroglycerine, clopidogrel, ASA, ephedrine, atropine	Recovery
Adachi et al. (12)	NR	NR	I	+/+	Atropine, adrenaline, nicorandil, noradrenaline, heparin	Recovery
Gao et al. (13)	NR	NR	I	NR	Steroid, epinephrine, promethazine, dopamine, norepinephrine, furosemide, deslanoside	Recovery, discharged after 11 d
Ricciardi et al. (14)	NR	NR	I	NR	Steroid, antihistaminic, statin	Recovery
Sato and Arai (15)	NR	NR	I	NR	Steroid, antihistaminic, nitroglycerin, ISDN, nicorandil, phenylephrine, ephedrine	Recovery
Ilgenli et al. (16)	NR	NR	I	NR	Steroid, antihistamines, ASA, clopidogrel, LMWH, CCB	Recovery
Kitulwatte et al. (17)	NR	NR	II	NR	Died	Died
Sequeira et al. (18)	NR	NR	I	−/+	Steroid, antihistamines, epinephrine, norepinephrine, ISDN	Recovery, discharged after 7 d
Biteker et al. (19)	NR	NR	I	NR	Steroid, antihistaminic	Recovery, discharged after 5 d
Murat et al. (20)	NR	NR	I	NR	Nitroglycerine, CCB	Recovery, discharged after 4 d
Yurtdaş et al. (21)	PCI	NR	I	+/NR	Steroid, antihistaminic, ASA, clopidogrel, heparin, nitroglycerine	Recovery, discharged after 5 d
Saleh et al. (22)	NR	NR	I	NR	Cardiopulmonary resuscitation, heparin, nitroglycerin, CCB	Recovery, discharged after 4 d
Barbarroja-Escudero et al. (23)	NR	NR	I	-/NR	Nitroglycerine	Recovery
Venkateswararao et al. (24)	NR	NR	I	NR/+	Steroid, antihistaminic, dual anti-platelets, statin, betablocker, LMWH, analgesics	Recovery, discharged after 4 d
Mitsis et al. (25)	NR	NR	I	NR	Steroid, antihistaminic, epinephrine, nitroglycerine, clopidogrel, ASA, CCB, LMWH	Recovery, discharged after 6 d
Absmaier et al. (26)	PCI	NR	II	−/+	Steroid, antihistaminic, ASA, heparin	Recovery
Çakmak and Keskin (27)	NR	NR	I	NR	Steroid, antihistaminic, adrenaline, nitroglycerine	Recovery, discharged after 3 d
Fujita et al. (28)	NR	NR	I	NR	Steroid, ephedrine, epinephrine, dopamine, ISDN	Recovery
Austin et al. (29)	NR	NR	I	NR	Steroid, antihistaminic, epinephrine, dopamine	Recovery
Ito et al. (30)	NR	yes	I	NR	Steroid, cardiopulmonary resuscitation, noradrenaline, nitroglycerin	Recovery, discharged after 11 d
Forlani et al. (31)	PCI	NR	II	NR/+	ASA, clopidogrel, statin	Recovery
Mota et al. (32)	NR	NR	I	−/+	Steroid, antihistaminic, epinephrine, ISDN, norepinephrine	Recovery

*ASA, acetylsalicylic acid; CCB, calcium channel blocker; DES, drug-eluting stent; IDT, intradermal test; ISDN, isosorbide dinitrate; LMWH, low molecular weight heparin; NR, not reported; PCI, percutaneous coronary intervention*.

## Discussion

Three variants of KS have been described: type I variant (coronary artery spasm, no risk factors for coronary heart disease); type II variant (previous history of coronary atherosclerosis); and type III variant (previous history of coronary stent implantation). The clinical features of KS are the simultaneous appearance of acute myocardial ischemia and acute allergic reactions (4). KS should be suspected for acute coronary syndrome with chest pain symptoms accompanied by allergic reactions. ECG, coronary angiography, cardiac markers, and tryptase help identify this syndrome (2). Coronary stents are an important means of treating ischemic heart disease, including bare metal stents with platforms, drug-eluting stents and bioabsorbable stents (33). Previous reports have demonstrated that all stent components, namely the stent platform with their metals (e.g., nickel, chromium, titanium, manganese, and molybdenum), polymer coatings, and released drugs are strong allergens which apply continuous, repetitive, persistent and chronic allergic irritation to the coronary intima (4, 34). All these types of stents are accompanied by rare but worrying stent thrombosis. Thus, stent thrombosis is primarily a manifestation of KS.

KS has a geographical distribution (4). Cephalosporin-induced KS is primarily distributed in Europe and Asia, especially Turkey and Japan. Our study confirmed that cefuroxime, ceftriaxone, and cefazolin were the most frequently reported cephalosporin antibiotics. The time from exposure to trigger to onset of KS was within 30 min in 80% of cephalosporin-induced KS patients. Our analysis found that many cases occurred during the perioperative period. The high incidence of cephalosporin-induced KS in the perioperative period may be due to the relatively frequent use of antibiotics in the perioperative period. Potential cross-reactions may occur with different β-lactams. Cephalosporins consist of a β-lactam ring and hydrothiazide ring. Two side chains (R1 and R2) distinguish the different cephalosporins. The cephalosporins cefuroxime, cefotaxime, cefpodoxime proxetil and ceftriaxone have methoxyimino groups on the R1 side chain, which may cause cross-reactions (35). We also cannot exclude the possibility of KS caused by the use of other concomitant drugs, such as metronidazole and rocuronium (36, 37).

Most studies suggest that the mechanism of KS is similar to allergic reactions. The main inflammatory cells, mast cells, interact with macrophages and T lymphocytes to cause the release of inflammatory mediators, including histamine, neutral proteases, arachidonic acid products, platelet activating factor and heparin, which lead to coronary spasm and coronary atherosclerosis plaque rupture or thrombosis in coronary stents (38).

The treatment of KS is extremely challenging because the heart symptoms and allergic symptoms must be considered simultaneously. There is no standard of treatment for KS, and treatment recommendations are based on the experience of case reports. Treatment of the allergic events with corticosteroids or H1 and H2 blockers alone eliminates symptoms in patients with type I variant (39, 40). The administration of calcium channel blockers and nitrates eliminates allergy-induced coronary artery spasms (36). For patients with type II and III variants, treatment of the acute myocardial ischemia and acute allergies is required (41). Epinephrine is a first-line drug for the treatment of severe allergies, but it should be used with caution in KS because it can aggravate myocardial ischemia and cause coronary artery spasm (42). Beta-blockers may exacerbate coronary spasm due to the lack of antagonism of alpha-adrenergic receptors (42). Opioids, such as morphine, codeine and pethidine, relieve the acute chest pain (43).

The prognostic factors for KS include the type of KS, the presence of complications, and the presence of allergens. Most patients with KS can expect a full recovery with appropriate treatment (43). Patients with type I KS have the best prognosis (44). Our research showed that cephalosporin-induced KS had a better prognosis. However, serious complications related to KS may occur, such as cardiac arrest and death.

## Conclusion

Clinicians and pharmacists should be aware of cephalosporin-induced KS to ensure the use appropriate therapeutic interventions and preventative measures. Antihistamines and steroids may be used to treat allergic reactions and nitrates and/or calcium channel blockers may be needed to treat coronary artery spasms.

## Data Availability Statement

The original contributions presented in the study are included in the article, further inquiries can be directed to the corresponding author.

## Author Contributions

WF and CW conceived the presented idea. WF, LS, ZD, WS, ZL, and CW wrote the manuscript. All authors discussed the results and contributed to the final manuscript.

## Funding

This study was supported by research grants from the National Natural Science Foundation of China (81900344).

## Conflict of Interest

The authors declare that the research was conducted in the absence of any commercial or financial relationships that could be construed as a potential conflict of interest.

## Publisher's Note

All claims expressed in this article are solely those of the authors and do not necessarily represent those of their affiliated organizations, or those of the publisher, the editors and the reviewers. Any product that may be evaluated in this article, or claim that may be made by its manufacturer, is not guaranteed or endorsed by the publisher.
